# Supplementary figures and images for: Enhanced biodiversity of gut flora and feed efficiency in pond cultured tilapia under reduced frequency feeding strategies
Source: PLoS One. 2020 Jul 23;15(7):e0236100. doi: 10.1371/journal.pone.0236100 (PMC7377384; doi:10.1371/journal.pone.0236100)

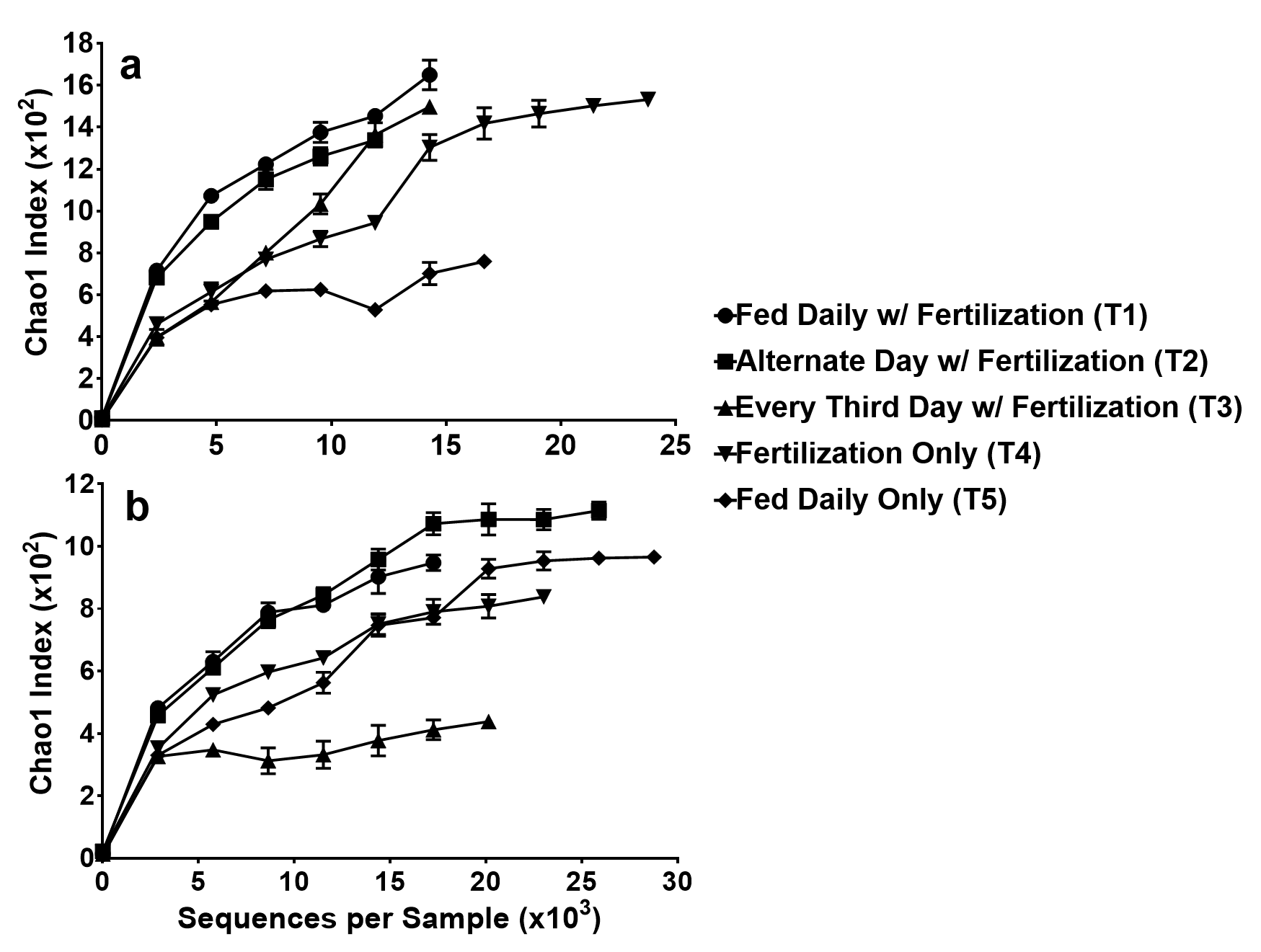

Supplement: S1 Fig — (a) 16S rRNA (prokaryotes); (b) 18S rRNA (eurkaryotes). (TIF) [file pone.0236100.s001.tif]
